# Comparison of Montreal Cognitive Assessment and Mini-Mental State Examination in Evaluating Cognitive Domain Deficit Following Aneurysmal Subarachnoid Haemorrhage

**DOI:** 10.1371/journal.pone.0059946

**Published:** 2013-04-03

**Authors:** George Kwok Chu Wong, Sandy Wai Lam, Adrian Wong, Karine Ngai, Wai Sang Poon, Vincent Mok

**Affiliations:** 1 Division of Neurosurgery, Department of Surgery, Prince of Wales Hospital, The Chinese University of Hong Kong, Hong Kong, China; 2 Division of Neurology, Department of Medicine and Therapeutics, Prince of Wales Hospital, The Chinese University of Hong Kong, Hong Kong, China; 3 Department of Psychological Studies, The Hong Kong Institute of Education, Hong Kong, China; University Of São Paulo, Brazil

## Abstract

**Objective:**

Cognitive deficits are common after aneurysmal subarachnoid haemorrhage (aSAH), and clinical evaluation is important for their management. Our hypothesis was that the Montreal Cognitive Assessment (MoCa) is superior to the Mini-Mental State Examination (MMSE) in screening for cognitive domain deficit in aSAH patients.

**Methods:**

We carried out a prospective observational and diagnostic accuracy study on Hong Kong aSAH patients aged 21 to 75 years who had been admitted within 96 hours of ictus. The domain-specific neuropsychological assessment battery, the MoCA and MMSE were administered 2–4 weeks and 1 year after ictus. A cognitive domain deficit was defined as a cognitive domain z score <−1.65 (below the fifth percentile). Cognitive impairment was defined as two or more cognitive domain deficits. The study is registered at ClinicalTrials.gov of the US National Institutes of Health (NCT01038193).

**Results:**

Both the MoCA and the MMSE were successful in differentiating between patients with and without cognitive domain deficits and cognitive impairment at both assessment periods. At 1 year post-ictus, the MoCA produced higher area under the curve scores for cognitive impairment than the MMSE (MoCA, 0.92; 95% CI, 0.83 to 0.97 versus MMSE, 0.77; 95% CI, 0.66 to 0.83, p = 0.009).

**Interpretation:**

Cognitive domain deficits and cognitive impairment in patients with aSAH can be screened with the MoCA in both the subacute and chronic phases.

## Introduction

Although aneurysmal subarachnoid haemorrhage (aSAH) accounts for only 3% of strokes, its profound consequences and unique window for intervention justify its classification as a separate entity [1]. Estimated independence in activities of daily living varies between 36% and 60% after aSAH [2], [3], and patients exhibit varying degrees of cognitive dysfunction [4], [5], [6]. Identifying cognitive dysfunction in aSAH patients is of paramount importance in patient management (i.e. medical treatment, cognitive rehabilitation and social arrangements).

A systematic review of aSAH cognitive dysfunction showed the main cognitive domain impairments to include memory, executive function and language, the prevalence of which ranges from 0% to 76% [3], [7]. The most common cognitive screening assessment tool used in neurosurgical clinics is the Mini-Mental State Examination (MMSE), which is quick (5–10 min) and easy to administer in a clinic setting. However, the MMSE was originally designed to screen for Alzheimer’s disease, and thus does not encompass all of the cognitive deficits that might occur following a stroke. It is particularly weak in its ability to measure executive functions, such as abstract thinking, judgment, problem solving and perception, all of which are relevant to the type of dementia associated with vascular disease. More recently, the Montreal Cognitive Assessment (MoCA) has also been used to screening for poststroke cognitive impairment [8]. The MoCA places greater emphasis on frontal executive function and attention tasks than does the more commonly used MMSE, and it may be more sensitive for the detection of non-Alzheimer’s disease dementia [9], [10]. Dong et al. further extended MoCA’s application during acute admission to predict vascular cognitive impairment 3–6 months after stroke [11]. Schweizer et al. subsequently found that when applied to 32 aSAH patients at a mean time of 29 months after aSAH, the MoCA was more sensitive to specific neurocognitive test scores than the MMSE, although they did not assess cognitive domain deficits [6]. The MoCA has also been shown to correlate with functional outcomes in aSAH patients at 3 months [12]. Godefroy et al., however, found that as a screening assessment for cognitive impairment, MoCA was not superior to MMSE with adjusted cut-off scores in subacute stroke patients [13].

Clinical disability and handicap scales are insensitive to poor neuropsychological outcomes. Accordingly, there is a well-recognised need to develop tests that are sensitive to the subtle but disabling effects of aSAH. The objective of the study reported herein was to collect additional data on evaluations of post-aSAH cognitive impairment and the theoretical advantages of using the MoCA in aSAH patients. Accordingly, we assessed the diagnostic accuracy of MoCA scores relative to the more commonly used MMSE scores in detecting post-aSAH cognitive impairment in the subacute and chronic phases of aSAH (i.e. 2–4 weeks and 1 year after ictus).

## Methods

This prospective observational four-centre study was carried out in Hong Kong. It is registered at ClinicalTrials.gov of the US National Institutes of Health (NCT01038193), and was approved by the Joint CUHK-NTEC Clinical Research Ethics Committee. It conformed to the Declaration of Helsinki, and written informed consent was obtained from all of the participants or their next of kin.

The patient inclusion criteria were: 1) spontaneous subarachnoid haemorrhage with angiography-confirmed aetiology of intracranial aneurysms; 2) hospital admission within 96 hours of ictus; 3) between 21 and 75 years of age; 4) speaker of Chinese (Cantonese); and 5) willing and able to provide informed consent (or availability of a person authorised to do so). The exclusion criteria were: a) a history of previous cerebrovascular or neurological disease other than unruptured intracranial aneurysm; b) a history of neurosurgery before ictus; and c) inability to cooperate in cognitive assessments (unable to obey commands).

Assessments were conducted 2–4 weeks (subacute phase) and 1 year (chronic phase) after ictus by one of two research assistants (psychology graduates) trained by a post-doctoral research psychologist. The cognitive assessments were carried out in a single session or in two sessions on consecutive days. The MMSE was administered before the cognitive domain-specific neuropsychological tests and the MoCA.

### Montreal Cognitive Assessment

The MoCA [9], [12] is a one-page, 30-point test that usually takes 15 minutes or less to administer and includes six subtests: visuospatial/executive functions, naming, attention, abstraction, recall and orientation [9], [12]. One point is added for participants with less than 12 years of education. The cut-off for mild cognitive impairment is 24/25 [9]. We recently reported the application of the Hong Kong version of MoCA in aSAH and neurosurgical patients following traumatic and spontaneous intracerebral haemorrhage [12], [14].

### Mini-Mental State Examination Chinese (Cantonese) Version

The MMSE [15] comprises seven sections (naming, orientation, registration, attention and calculation, recall, praxia, and language). Its maximum total score is 30, and the test can usually be completed in 10 minutes or less. The Cantonese version has been validated in a population of dementia patients, for whom the optimal cut-off was 19/20 [16].

The battery of cognitive assessments used in this study was previously applied in a local Chinese population [17]. Its selection was based on (a) its efficacy in previous cognitive studies in local Chinese patients and standard cognitive tests validated in a Cantonese-speaking population, and (b) its balanced range of tests covering verbal and visuospatial memory, attention and working memory, executive functions, psychomotor speed and language. This battery included the following.

### Verbal Memory Domain

#### i.) Hong Kong List Learning Test (HKLLT) [18]

The HKLLT is based on the California Verbal Learning Test, which is regularly used in vascular cognitive impairment studies. It is a verbal learning and memory test that consists of two 16-word lists with three learning trials: immediate recall, 10-minute delayed recall and 30-minute delayed recall and recognition. The HKLLT has been validated in both normal and pathological local populations [18].

### Visuospatial Skill and Memory Domain

#### i.) The Rey Osterrieth Complex Figure Test

The Rey Osterrieth Complex Figure Test is commonly used to assess visuospatial construction skills and visuospatial memory [19].

### Attention and Working Memory Domain

#### i.) The verbal and visual digit span forward and backward tests from the Chinese Wechsler Memory Scale-Third Edition [20] are used to evaluate simple attention and working memory

Verbal and visual spans have been used as donor scales for composite psychometric measures.

### Executive Function and Psychomotor Speed Domain

#### i.) Symbol-Digit Modalities Test [21]

This brief, easy-to-administer, timed coding test is a variant of the Digit-Symbol Coding Task in the Wechsler Adult Intelligence Scale-Third Edition.

#### ii.) Color Trails Test (CTT)

This test originates from the Trail Making Test (TMT), which is used in the timed assessment of psychomotor speed and executive functions. As it features coloured numbers rather than the English alphabet, the CTT is considered to be an acceptable cultural substitute for the original TMT and has been shown to have similar psychometric properties [22].

#### iii.) Animal fluency

This test requires subjects to generate as many animal names as possible in one minute. It is a simple timed test that measures both speed and activation and such executive processes as clustering, set-shifting and retrieval [18], [23].

### Language Domain

#### i.) modified Boston Naming Test (mBNT) [24]

The Boston Naming Test is the most frequently used confrontation naming test in assessing language. In this study, we used the mBNT, a validated modified version that contains 15 stimuli appropriate for use in Chinese cultures [18].

Cognitive domain scores were computed by averaging the z scores of the respective test measures derived from established age- and education-matched norms. A cognitive domain deficit was defined as a cognitive domain z score <−1.65 (below the fifth percentile). Cognitive impairment was defined as two or more cognitive domain deficits [25].

### Statistical Analysis

Statistical analyses were generated using SPSS for Windows Version 15.0 (SPSS Inc., Chicago, IL, USA) and MedCalc Version 12.2.1.0. Categorical data are presented herein as numbers (percentages) and numerical data as medians and interquartile ranges (IQR), unless otherwise specified. In the assessments of cognitive impairment, a difference with a P value of less than 0.05 was regarded as statistically significant. For the secondary analyses of individual domain deficits, a difference with a P value of less than 0.01 (to account for multiple comparisons) was regarded as statistically significant.

Receiver operating characteristic (ROC) curves were constructed to examine the ability to distinguish post-aSAH cognitive impairment and the five individual domain deficits (the verbal memory, visuospatial skill and memory, attention and working memory, executive function and psychomotor speed, and language domains) at both 2–4 weeks and 1 year. The area under the curve (AUC) was calculated for each of the aforementioned ROC curves with a 95% confidence interval (95% CI). The AUC represents the probability that the score of a normal sample will be higher than that of an abnormal sample when one sample is drawn from a truly normal population and the other from a truly abnormal population. The statistical significances of the differences between the correspondingly paired MoCA and MMSE AUCs of cognitive impairment and the five individual domain deficits were then assessed using the nonparametric approach adopted by Delong et al. [26]. For the MoCA and MMSE ROC curves for cognitive impairment at 2–4 weeks and 1 year, cut-off values were derived at the ROC coordinate points, where both sensitivity and specificity were optimised using the maximised Youden Index (J) [27]. The Youden Index is defined as sensitivity (true positive)+specificity (true negative) –1 [28]. Complete separation of the marker values for abnormal and normal populations results in J = 1, whereas their complete overlap produces 0. The discriminant indices (sensitivity, specificity, positive and negative predictive values, and diagnostic accuracy [% correctly classified]) of cognitive impairment at the optimal cut-offs were calculated for MoCA and the MMSE at the two assessment points.

The reproducibility of the assessments was not assessed because it was not the focus of the study, and would have been impractical considering the study’s design (possible rehearsal effects and additional inconvenience to patients and their families).

## Results

Seventy-four and 80 patients completed all of the cognitive assessments at 2–4 weeks and 1 year, respectively. The patient profiles are presented in Tables 1 and 2. No adverse events were reported in relation to the assessments.

**Table 1:** Patient profile.

	Patients completing assessment at 2–4 weeks	Patients completing assessments at 1 year
	(n = 74)	(n = 80)
Age, median (IQR)	58(49–66)	52(47–61)
Female (%)	50(68)	55(69)
Hypertension (%)	27(37)	27(34)
Smoker (%)	26(35)	22(28)
WFNS Grade		
I	48(65)	45(56)
II	15(20)	21(26)
III	4(5)	1(1)
IV	6(8)	9(12)
V	1(1)	4(5)
Location of aneurysm		
ICA other than PComA	11(15)	16(20)
PComA	13(18)	16(20)
Anterior cerebral artery	24(32)	26(33)
Middle cerebral artery	18(24)	20(25)
Posterior circulation	8(11)	12(15)
Aneurysm treatment		
Coiling	36(49)	43(54)
Clipping	38(51)	27(46)
		
Delayed cerebral Infarction	3(4)	10(13)
Clinical deterioration due to DCI	10(14)	7(9)
mRS		
0	9(12)	22(28)
1	4(5)	12(15)
2	28(38)	33(41)
3	15(20)	11(14)
4	17(23)	1(1)
5	1(1)	1(1)

ICA: internal carotid artery; PComA: posterior communicating artery; DCI: delayed cerebral ischemia; mRS: modified Rankin Scale; WFNS: World Federation of Neurosurgical Societies.

**Table 2:** Neuropsychological profile of subacute and chronic phase participants.

	Patients completing assessments at 2–4 weeks	Patients completing assessments at 1 year
	B (n = 74)	C (n = 80)
Cognitive impairment	12(16%)	12(15%)
*Cognitive domain deficit*		
Verbal memory	12(16%)	6(8%)
Visuospatial skill and memory	16(22%)	13(16%)
Attention and working memory	4(5%)	6(6%)
Executive function and psychomotor speed	14(19%)	11(14%)
Language	6(8%)	7(9%)

**Figure 1:**
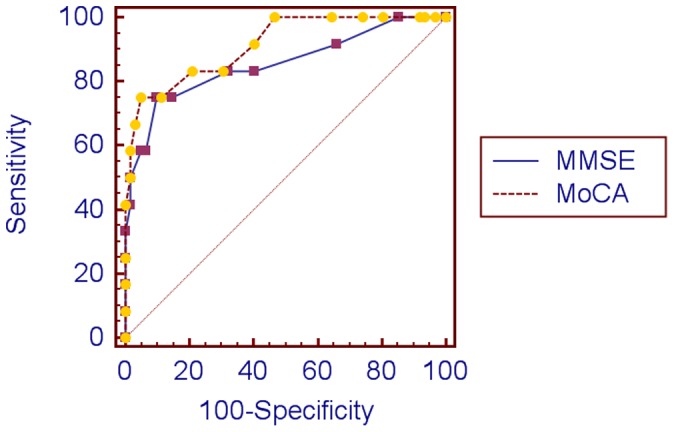
Receiver operating characteristic curves of cognitive impairment at 2–4 weeks.

**Figure 2:**
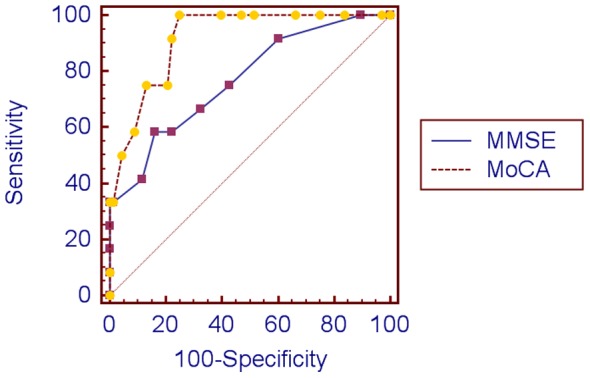
Receiver operating characteristic curves of cognitive impairment at 1 year.

Table 3 shows that both the MoCA and the MMSE were able to differentiate between patients with and without cognitive domain deficits and cognitive impairment. At 2–4 weeks, the two screening methods produced similar AUCs for both cognitive domain deficits and cognitive impairment (Table 4 and Figure 1). At 1 year, the MoCA achieved significantly higher AUCs than the MMSE for cognitive impairment, although not for single domain deficits (Table 5 and Figure 2).

**Table 3:** Discriminant indices of the Montreal Cognitive Assessment and Mini-Mental State Examination in detecting cognitive impairment.

	Cuff-off value	Sen (95% CI)	Spec (95% CI)	PPV (95% CI)	NPV (95% CI)	Diagnostic Accuracy
At 2–4 weeks						
MoCA	17/18	75(43–95)	95(87–99)	75(41–95)	95(87–99)	92%
MMSE	23/24	75(43–95)	90(80–96)	60(32–84)	95(86–99)	88%
At 1 year						
MoCA	21/22	100(74–100)	75(63–85)	41(24–61)	100(93–100)	85%
MMSE	23/24	58(28–85)	84(73–92)	39(17–64)	92(82–97)	80%

Sen: Sensitivity; Spec: Specificity; PPV: Positive Predictive Value; NPV: Negative Predictive Value.

MoCA: Montreal Cognitive Assessment.

MMSE: Mini-Mental State Examination.

**Table 4:** Areas under the curve (AUCs) for the Mini-Mental State Examination and Montreal Cognitive State Examination in assessing cognitive deficits at 2–4 weeks.

	MMSE	MoCA	
	AUC (95% CI)	AUC (95% CI)	P-value
Cognitive impairment	0.86 (0.75–0.93)	0.91 (0.83–0.97)	0.324
Executive function and psychomotor speed	0.76 (0.65–0.86)	0.81 (0.71–0.90)	0.384
Verbal memory	0.62 (0.50–0.73)	0.68 (0.56–0.78)	0.389
Visuospatial memory and skill	0.84 (0.74–0.92)	0.90 (0.80–0.96)	0.291
Attention and working memory	0.97 (0.89–0.99)	0.88 (0.78–0.94)	0.349
Language	0.96 (0.88–0.99)	0.97 (0.90–1.00)	0.535

* P<0.05;

** P<0.01.

AUC: Area under curve.

MMSE: Mini-Mental State Examination.

MoCA: Montreal Cognitive Assessment.

**Table 5:** Areas under the curve (AUCs) for the Mini-Mental State Examination and Montreal Cognitive State Examination in assessing cognitive deficits at 1 year.

	MMSE	MoCA	
	AUC (95% CI)	AUC (95% CI)	P-value
Cognitive impairment	0.77 (0.66–0.83)	0.92 (0.83–0.97)	0.009**
			
Executive function and psychomotor speed	0.74 (0.63–0.83)	0.86 (0.77–0.93)	0.044*
Verbal memory	0.86 (0.76–0.93)	0.93 (0.85–0.97)	0.052
Visuospatial memory and skill	0.89 (0.79–0.95)	0.85 (0.76–0.92)	0.539
Attention and working memory	0.65 (0.53–0.75)	0.79 (0.69–0.88)	0.099
Language	0.75 (0.64–0.84)	0.91 (0.82–0.96)	0.018*

* P<0.05;

** P<0.01.

AUC: Area under curve.

MMSE: Mini-Mental State Examination.

MoCA: Montreal Cognitive Assessment.

In screening for cognitive impairment defined as two or more cognitive domain deficits, the optimal cut-offs for the MoCA and MMSE were ≤18 and ≤24, respectively, at 2–4 weeks and ≤22 and ≤24, respectively, at 1 year (Table 3), with diagnostic accuracy (% correctly classified) ranging from 80% to 92%.

## Discussion

The results of this study demonstrate the accuracy of the MoCA in the diagnosis of post-aSAH cognitive domain deficits and cognitive impairment at both the subacute and chronic stages. Further, they show the MoCA to be superior to the MMSE in diagnosing post-aSAH cognitive impairment at the chronic stage.

Godefroy et al. recently assessed 95 patients (infarct: n = 88; haemorrhage: n = 7) to compare the efficacy of the MoCA and MMSE in detecting poststroke cognitive impairment, as determined by a neuropsychological battery, at a mean of 24 days poststroke [13]. Both demonstrated good ability to discriminate between impaired and nonimpaired cognitive status (AUCs >0.88). The MoCA performed similarly to the MMSE at an adjusted cut-off score of ≤20. These researchers’ results were similar to our observations in aSAH patients in the subacute phase.

Schweizer et al. reported the first case series using the MoCA in aSAH patients. They recruited 32 aSAH patients with favourable neurological outcomes (31 good recoveries and 1 moderate disability on the Glasgow Outcome Scale) [6]. Neurocognitive assessments were performed at least 6 months after aSAH. They found that the MMSE total score did not correlate with any neurocognitive test score except the Boston Naming Test score for language. The MoCA total score, in contrast, was correlated with the Wisconsin Card Sorting test score for executive function, Boston Naming Test score for language and California Verbal Learning test score for verbal learning and memory. They did not evaluate the relationship between the MoCA and cognitive domain deficits. The results of the current study confirm the suggested superiority of the MoCA over the MMSE in screening post-aSAH cognitive domain deficit and cognitive impairment at 1 year using a domain-specific neuropsychological assessment battery.

Our optimal cut-offs (≤18 at 2–4 weeks and ≤24 at 1 year) were lower than the original cut-off (≤25) proposed by Nasreddine et al. [9]. One major difference between the two studies is that we were screening for cognitive impairment characterised by two or more cognitive domain deficits, whereas their focus was mild cognitive impairment. It is also possible that different language versions of the screening tools may yield different cut-off scores for the same disease entity [29] and that the nature of the disease may differ across studies.

It is not known why the MoCA performed better than the MMSE in detecting cognitive impairment at the chronic stage but not the subacute stage in this study. We speculate that diffuse brain injuries significantly overshadow focal brain injuries in the manifestation of cognitive impairment at the subacute stage, whereas the reverse occurs at the chronic stage. It is recommended that biomarker studies be considered in future to investigate this interesting phenomenon.

Passier et al. recently reported domain-based cognitive functioning at 3 months post-aSAH to be the most relevant independent predictor of health-related quality of life at 1 year post-aSAH [30]. They also concluded that it is important to screen all patients because reduced health-related quality of life is not limited to patients discharged to a rehabilitation facility. The urgency of the need to find an optimal screening test for aSAH patients cannot be understated.

MoCA’s feasibility has also been evaluated in a stroke clinical trial [31]. Of those patients who survived to 3 months, the MoCA was completed by 87% of those with mild stroke, 79% with moderate stroke and 67% with severe stroke on admission. The MoCA scale was shown to have a high degree of internal consistency, with all items loading onto a single factor that accounted for nearly half the variance. These findings further support the clinical and research application of the MoCA as a screening test for cognitive impairment in aSAH patients.

### Limitations of This Study

This study had several limitations. First, although the domain-based neuropsychological battery we used has been validated in a Chinese population with established norms, it is possible that it was insufficiently sensitive and comprehensive to measure subtle cognitive changes in patients with milder occurrences of the disease [32]. Second, the cognitive domain was treated as a unitary construct rather than as a collection of different cognitive abilities. Third, undergoing the battery of cognitive assessments in addition to the MMSE and MoCA is tiring, and fatigue may have affected patients’ performance, although to compensate we offered a 5- to 10-minute rest in the middle of the assessment sessions or divided the assessments over two consecutive days. Finally, the study’s aim was to assess the cognitive profile of aSAH patients, and thus its results may not be applicable to other stroke subtypes.

### Conclusion

We have shown here that (1) the MoCA is able to identify cognitive impairment and cognitive domain deficits in the subacute and chronic phases of aSAH and (2) is superior to the MMSE in detecting cognitive impairment at 1 year following ictus.
